# An upward trajectory of genomic publications from Africa: cautious optimism for a turning tide

**DOI:** 10.1017/gheg.2018.14

**Published:** 2018-10-08

**Authors:** Michèle Ramsay

**Affiliations:** South African Research Chair in Genomics and Bioinformatics of African Populations and The Sydney Brenner Institute for Molecular Bioscience, Faculty of Health Sciences, University of the Witwatersrand, South Africa

Africa has been a favoured destination for helicopter research for many decades with little regard to building and strengthening research capacity on the continent. Those were the days of general verbal consent, incentivisation and sometimes coercion. More recently, we have witnessed a global ground swell of support for community engagement and public education as a prelude to ethical genomic research [1–4]. Yet the lack of vocabulary for genetics and genomics, especially in isolated and vulnerable communities, means that there is a need to take more time to develop thoughtful, culturally attuned and creative explanations and analogies to solicit informed consent. This is especially challenging when researchers request broad consent for the future use of data and samples. Thus, in such cases, the governance of data and samples usually includes local ethics review committees that serve as the third-party protector from harm to research participants.

Notwithstanding the challenges of performing and funding ethically sound research in Africa, it is encouraging to observe that genomic publications with a connection to Africa have increased significantly over the past 20 years and now make up almost 2% of all publications in PubMed for the terms ‘genomics’, ‘GWAS’ and ‘pharmacogenomics’ (Table 1). What is the story behind the finding from this simple PubMed search, counting the number of publications in four consecutive 5-year periods? The sceptics would suggest that the research is still primarily externally driven and performed outside the continent, leveraging nominal collaborations in an unequally resourced set of environments. However, through initiatives such as the Human Heredity and Health in Africa Consortium (H3Africa) [5] and funding opportunities that mandate partnerships with low-income and middle-income countries in order to promote global health research, those more optimistic would suggest that the tide is turning.

**Table 1.** PubMed listed publications and percentage of papers with an African connection^a^

	Genomics			GWAS			Pharmacogenomics		
Publication date	Total	Africa	% Africa	Total	Africa	% Africa	Total	Africa	% Africa
1998–2002	8279	29	0.35	86	1	1.16	1837	15	0.82
2003–2007	28 432	166	0.58	643	8	1.24	4417	26	0.59
2008–2012	48 979	359	0.73	12 189	109	0.89	5881	56	0.95
2013–2017	90 421	1682	1.86	18 883	314	1.66	8123	160	1.97
Total	176 111	2236	1.27	31 801	432	1.36	20 258	257	1.27

a Method: PubMed search with the terms ‘genomics’; ‘Africa genomics’; ‘GWAS’; ‘Africa GWAS’; ‘pharmacogenomics’; and ‘Africa pharmacogenomics’. Five-year period year/01/01 to year/12/31.

Much has been written about the extraordinary value of data, especially big genomic and phenotype data, in finding connections that may have implications for health [6]. Initiatives like the UK biobank with over 500 000 participants (http://www.ukbiobank.ac.uk/), 23andme (https://www.23andme.com/) with data on over 5 million people, and several other national and transnational consortia make this possible. By design, they often have no African participants or have only a small proportion of people with African ancestry. Of those, a minority originate directly form the African continent that is home to 1.2 billion people (~16% of the world population) [7].

What would it take to enable genomic research and the possibility of a public health approach to precision medicine in Africa for the benefit of its inhabitants, its diaspora populations and the world at large? Building on the writings of others [8], I would suggest five broad areas of focus to build the enablers for the process. First and foremost, we need to collect good data across countries and communities to measure the basics like births, deaths, causes of death and infections. This will make it possible to identify the main contributors to morbidity and mortality and to develop priorities. For example, in a country where neonatal deaths are common, one would focus on prevention of infection and basic postnatal care. When older adults die during their economically active years from stroke and the complications of diabetes, the focus could turn to education about lifestyle and national policies to address modifiable risk factors. Secondly, capturing the data in carefully constructed and robust electronic formats that reach the most remote of locations will provide hard evidence for national prioritisation and planning. This is becoming increasingly possible with greater access to electrical and solar power and the ubiquitous use of mobile devices. Thirdly, there is a need for basic enabling infrastructure and this would include stable computer networks, diagnostic laboratory infrastructure and affordable devices, and an educational system that invests in building and supporting research infrastructure. Research has a context and it is important to solve local problems and develop interventions appropriate to a location and culture. Fourthly, none of this would be possible without enough people with the relevant skills to perform all of the above. Africa has a vast untapped talent base. It is essentially a matter of scale and opportunity. How do we train and retain more IT specialists, technicians, field workers, doctors and nurses, computer scientists, genomic researchers and project leaders? This brings me to the last point which is political will. It does not need excessive resources to do the basics, it requires leadership to invest in the future. A clear vision and set of national and regional implementation strategies could propel Africans to a healthier future.

It is encouraging that there has been more research with an African connection in the field of genomics over the past 5 years. The trend augers well and small steps with high-impact discoveries do make a difference.

## References

[1] de Vries J, Littler K, Matimba A, McCurdy S, Ouwe Missi Oukem-Boyer O, Seeley J and Tindana P (2016) Evolving perspectives on broad consent for genomics research and biobanking in Africa. Global Health, Epidemiology and Genomics 1, e13. 10.1017/gheg.2016.5 PMC5870421 29868205

[2] Tindana P, Campbell M, Marshall P, Littler K, Vincent R, Seeley J, de Vries J, Kamuya D and H3Africa Community Engagement Working Group (2017) Developing the science and methods of community engagement for genomic research and biobanking in Africa. Global Health, Epidemiology and Genomics 2, e13. 10.1017/gheg.2017.9 PMC5732574 29276620

[3] Claw KG, Anderson MZ, Begay RL, Tsosie KS, Fox K, Garrison NA and Summer internship for INdigenous peoples in Genomics (SING) Consortium (2018) A framework for enhancing ethical genomic research with Indigenous communities. Nature Communications 9, 2957. 10.1038/s41467-018-05188-3 PMC6063854 30054469

[4] Samuel GN and Farsides B (2018) Genomics England's implementation of its public engagement strategy: blurred boundaries between engagement for the United Kingdom's 100,000 Genomes project and the need for public support. Public Understanding of Science 27, 352–364. 29241419 10.1177/0963662517747200 PMC5841566

[5] H3Africa Consortium (2014) Research capacity. Enabling the genomic revolution in Africa. Science 344, 1346–1348. 24948725 10.1126/science.1251546 PMC4138491

[6] Manolio T (2017) In retrospect: a decade of shared genomic associations. Nature 546, 360–361. 28617469 10.1038/546360a

[7] Popejoy AB and Fullerton SM (2016) Genomics is failing on diversity. Nature 538, 161–164. 27734877 10.1038/538161a PMC5089703

[8] Dowell SF, Blazes D and Desmond-Hellmann S (2016) Four steps to precision public health. Nature 540, 189–191.

